# *Lopadostoma*, *Oligostoma*, and some *Rosellinia* specimens from the herbarium of the Swiss Federal Institute of Technology (ZT): the value of early fieldwork and the importance of keeping fungal collections

**DOI:** 10.1186/s40529-023-00404-w

**Published:** 2023-11-14

**Authors:** Liliane E. Petrini

**Affiliations:** Via al Perato 15c, Breganzona, Switzerland

**Keywords:** Collections, Field work, Herbarium, Lopadostomataceae, Mycology, New taxon, Taxonomy, Xylariaceae

## Abstract

**Background:**

Morphology, hosts, and collecting sites of fungi assessed from herbarium material of special interest deserve to be brought to the attention of mycologists.

**Results:**

Specimens of *Lopadostoma* and *Oligostoma* deposited at ZT are briefly described and listed to expand the knowledge about their distribution. Three yet unmentioned *Rosellinia* collections are reported. One could be identified as *R. mastoidiformis*, a second as *R. neblina*; both are known only from the type collections. The third one seems to be a yet undescribed taxon and is formally described as *R. schueppii.*

**Conclusions:**

These observations emphasize the importance of keeping fungal collections and highlight the importance of field work and contributions by early mycologists.

## Background

Two recent phylogenetic and taxonomic revisions of *Lopadostoma* Nitschke (Jaklitsch et al. 2014) and *Xylariaceae* with remarkable germ loci (Voglmayr et al. 2022) have provided important contributions toward solving the puzzle of Xylariaceous genera and stimulated me to check my notes on *Lopadostoma* and other similar specimens in the Herbarium ZT I examined long time ago.

In the past, small-spored *Lopadostoma* collections from *Fagus sylvatica* were named *L. turgidum* (Pers.) Trav., whereas larger spored ones were labelled as *L. gastrinum* (Fr.) Trav. Presently, accepted *Lopadostoma* species on *Fagus* are *L. fagi* Jaklitsch, J. Fourn. & Voglmayr and *L. turgidum.* They are clearly distinct, the ascospores of *L. turgidum* being longer and wider with broadly rounded ends and having a unilateral germ slit, compared to those of *L. fagi* with narrowly rounded ends and a circumferential germ slit (Jaklitsch et al. 2014). *Lopadostoma gastrinum* occurs mainly on *Ulmus* and other not quercicolous hosts, whereas *L. quercicola* Jaklitsch, J. Fourn. & Voglmayr is restricted to *Quercus* spp. *Lopadostoma gastrinum* and *L. quercicola* have ascospores of comparable size with a circumferential germ slit but molecular phylogeny separates them in two distinct clades (Jaklitsch et al. 2014). Subtle stromal variations are often missed by microscopic examination (Jaklitsch et al. 2014).

Among the *Rosellinia* collections I have studied and are deposited at the Swiss Federal Institute of Technology (ZT), three *Rosellinia* specimens, two collected in South Africa and one in Puerto Rico are also worth mentioning. Two are similar to species known only from the type collections, and one seems to be undescribed.

In this study I provide short descriptions and a list and of *Lopadostoma* and *Oligostoma* specimens deposited at ZT based on notes taken over 30 years ago and describe the three *Rosellinia* collections to expand the knowledge about their distribution. The aim of this work is also to emphasize the importance of keeping and studying herbarium fungal collections and to value the field work and contributions by early mycologists.

## Materials and methods

Microscopy was carried out as described by Petrini (2013). Ascospores were mounted and measured in water, ascus apical plugs in Melzer’s reagent.

Spore measurements [minimum (min), maximum (max), mean, standard deviation(sd)] are presented as [min–] mean (sd) [–max], length x width. Other measurements indicate the range of minimum and maximum values. A k^th^-nearest-neighbour discriminant analysis used ascospore size, germ slit morphology, and host as variables to identify specimens (Table 1). Biplots were used to display associations between characters and taxa as well as similarities among taxa. A biplot displays simultaneously the observations and the relative positions of the variables used to characterize samples. Marker symbols (points) are displayed for observations, and arrows are displayed for variables. Observations are projected on two dimensions such that the distance between the observations is approximately preserved. All analyses and graphical displays were carried out using Stata (StataCorp,College Station, Texas, USA) version 18.

**Table 1:** k^th^-nearest-neighbor discriminant analysis using spore length and width, germ slit morphology, and host as discriminant variables – classification summary [counts (%)]

Species	*L. fagi*	*L. gastrinum*	*L. quercicola*	*L. turgidum*	Total counts
***L. fagi***	120 (92.3)	0 (0.0)	0 (0.0)	10 (7.7)	130 (100.0)
***L. gastrinum***	0 (0.0)	111 (92.5)	9 (7.5)	0 (0.0)	120 (100.0)
***L. quercicola***	0 (0.0)	4 (2.9)	136 (97.1)	0 (0.0)	140 (100.0)
***L. turgidum***	3 (1.9)	0 (0.0)	0 (0.0)	157 (98.1)	160 (100.0)
**Total counts**	123 (22.4)	115 (20.9)	145 (26.4)	167 (30.4)	550 (100.0)

## Results

Based on ascospore measurements, specimens on *Fagus* could be attributed as expected either to *L. turgidum* or *L. fagi*, whereas those on *Quercus* to *L. quercicola*, and three specimens on *Ulmus*, one on *Acer campestris*, and one on *Abies* (tentative host) to *L. gastrinum* (Table 1). The *Anthostoma rhenanum* Fuckel ex. Sacc. specimens could be identified as *Oligostoma insidiosum* (P. Crouan & H. Crouan) Voglmayr, J. Fourn. & Jaklitsch.

**Fig. 1:**
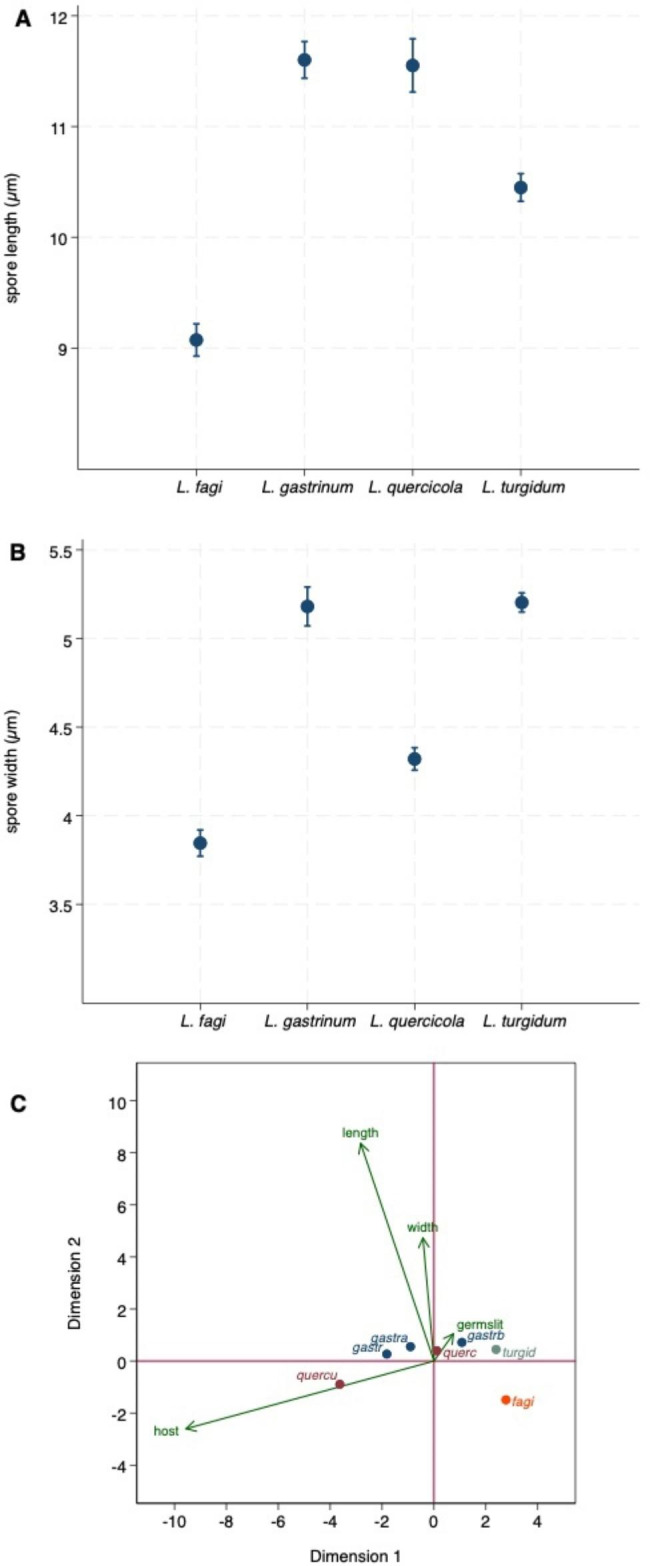
** A** 95% Confidence interval of ascospore length. **B** 95% Confidence interval of ascospore width. **C** Biplots showing associations between characters and taxa and similarities among taxa. fagi: *L. fagi*; gastr: *L. gastrinum* on *Ulmus*; gastra: *L. gastrinum* on *Acer;* gastrb: *L. gastrinum* on *Abies;* length: ascospore length; querc: *L. quercicola* on Quercus; quercu: *L. quercicola* on undetermined host, probably on *Quercus*; turgid: *L. turgidum*; width: ascospore width. For details about the biplot presentation see Material and Methods section

Figure 1A, B display the 95% confidence intervals of ascospore length and width showing small, but statistically significant differences. The discriminant analysis (Table 1) correctly identified 98% of *L. turgidum* measurements, 97% of the *L. quercicola*, and 92% of *L. fagi*, with 8% of the *L. fagi* measurements identified as belonging to *L. turgidum*. For *L. gastrinum* 92.5% of the data were correctly assigned, whereas 7.5% were classified as *L. quercicola*.

*Lopadostoma fagi, L. quercicola*, and *L. gastrinum* have ascospores with a circumferential germ slit. In *L. fagi*, the ascospores are smaller than in the other two species. *Lopadostoma quercicola, L. gastrinum*, and *L. turgidum* have overlapping ascospore sizes; the former two differ from each other by host and molecular phylogeny. *L. turgidum* has ascospores with a unilateral germ slit and fruits on a different host. The graph of the biplot analysis reflects these differentiating characters (Fig. 1C; variance explained by the two dimensions: 98%). Host and spore size distinguish *L. fagi* from *L gastrinum* and *L. quercicola*, whereas spore size and germ slit from *L. turgidum*. Host and germ slit separate *L. turgidum* from *L. gastrinum* and *L. quercicola*. The unconfirmed host of some *L. quercicola* specimens separates them from the other *L. quercicola* on *Quercus spp.* Only the host distinguishes *L. quercicola* and *L. gastrinum* on *Abies* (?), *Acer*, and *Ulmus*.

## Descriptions

**Fig. 2:**
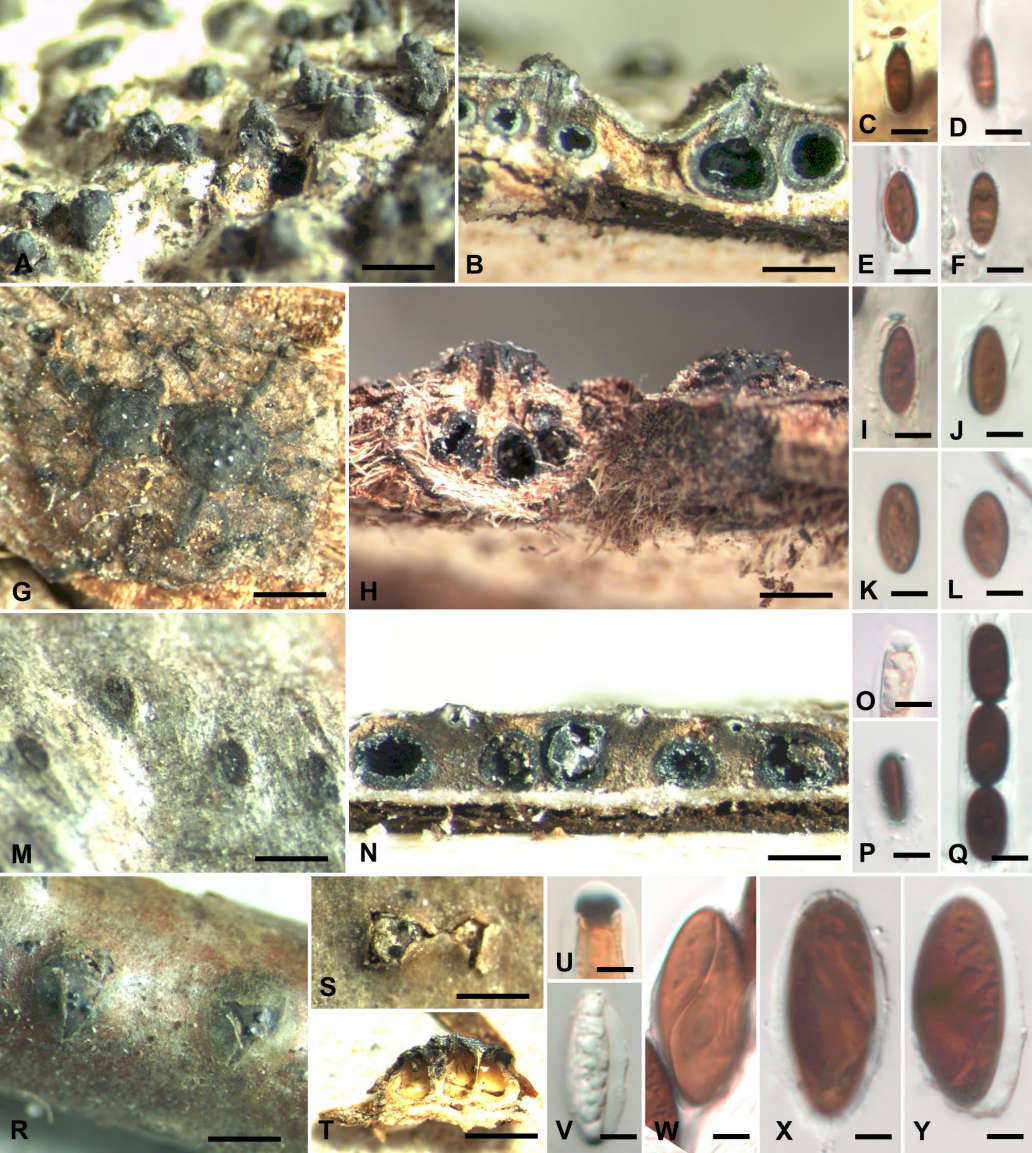
*Lopadostoma fagi* **A** Ostioles emerging on host surface. **B** Cross sections of stromata (ZT Myc 0081700). **C** Ascus apical plug in Melzer’s reagent (ZT Myc 0081724). **D** Ascospore showing germ slit (ZT Myc 0087124). **E, F** Ascospores (ZT Myc 0081700). *Lopadostoma gastrinum* **G** Ostiolar discs on host surface (ZT Myc 0081702), **H** Cross section of stroma and stroma case with ostiolar disk on the right (ZT Myc 0081703). **I** Ascus apical plug in Melzer’s reagent (ZT Myc 0081703). **J-L** Ascospores (ZT Myc 0081702). *Lopadostoma turgidum* **M** Ostiolar dics on host surface. **N** Cross section of stromata. **O** Ascus apical plug in Melzer’s Reagent (M-O ZT Myc 0081725). **P** Ascospore showing germ slit (ZT Myc 081701). **Q** Ascospores (ZT Myc 0081725). *Oligostoma insidiosum* **R, S** Ostiolar discs erumpent from bark (R ZT Myc 0081723, S ZT Myc 0081721). **T** Cross section of stroma (ZT Myc 0081718). **U** Ascus apical plug in Melzer’s Reagent (ZT Myc 0081719). **V** Immature ascospores (ZT Myc 0081721). **W** Ascospore showing germ slit (ZT Myc 0081718). **X, Y** Ascospores (ZT Myc 0081720). Scale bar: **T =** 2 mm. **A, G, H, R, S** = 1 mm. **B, M, N** = 0.5 mm. **C-F, I-L, O-Q, U-Y** = 5 μm

**Fig. 3:**
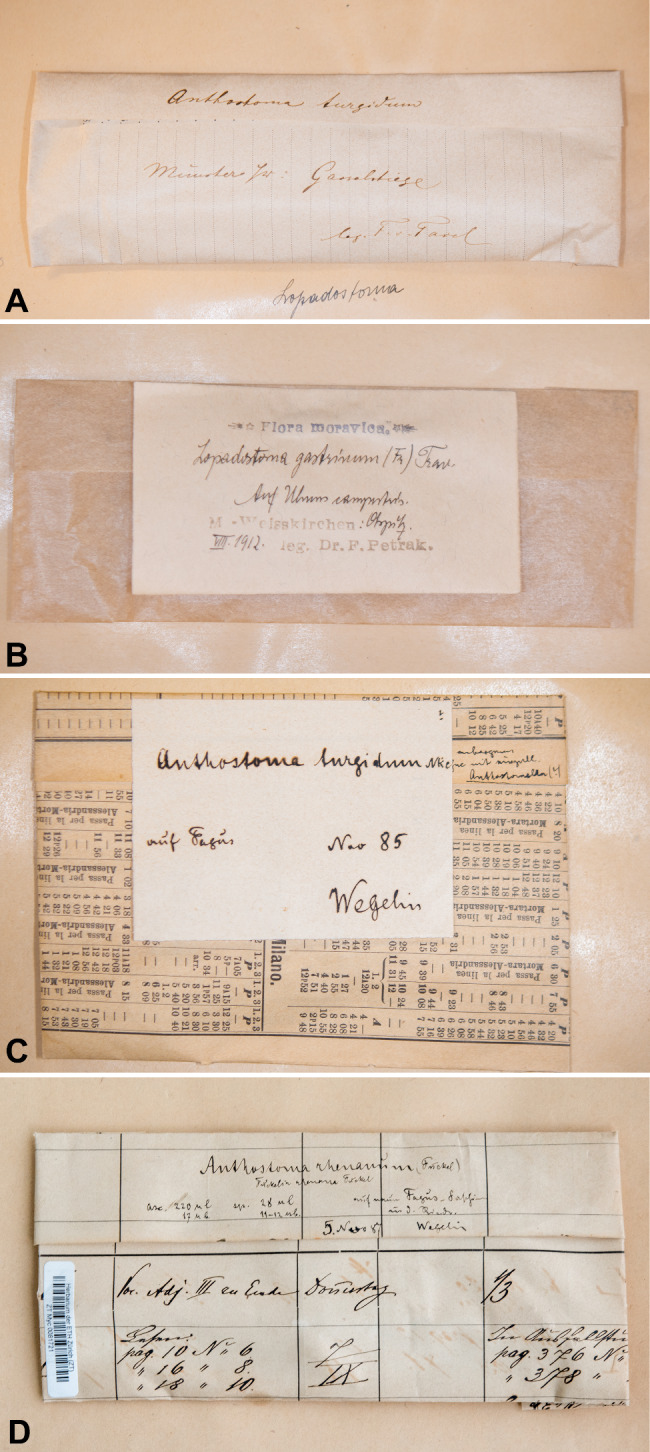
Labels of some herbarium specimens. **A** *Anthostoma turgidum*, leg. F. v. Tavel (ZT Myc 0081856, *Lopadostoma fagi*). **B** Aug. 1912, leg. F. Petrak, *L. gastrinum* (ZT Myc 0081855). **C** *Anthostoma turgidum*, Nov. 1885, leg. Wegelin (ZT Myc 0081853, *L. turgidum*). **D** *Anthostoma rhenanum*, 05. Nov. 1887, leg. Wegelin (ZT Myc 0081721, *Oligostoma insidiosum*)

***Lopadostoma fagi*** Jaklitsch, J. Fourn. & Voglmayr, Persoonia 32: 63 (2014). Figure 2 A–F, Fig. 3A.

For a complete description and illustrations see Jaklitsch et al. (2014).

Stromata 1–2 mm wide, semiglobose to flatly pulvinate, button like, black, with central disk, laterally encasing the perithecia, stromatic line 25–50 μm thick, singly, crowded. Ostiolar disc 0.2–0.8 mm in diam., raising up to 0.1–0.4 mm above the bark, black, warty from perforating ostioles. Entostroma between perithecia brown, below cream to white if developed. Perithecia 250–775 μm wide, 300–725 μm high excl. ostioles (n = 30), 4–11 per stroma. Ostioles 300–625 μm long, convergent into disc. Asci total length 100–119 μm, spore bearing part 72–78 μm, stipe 24–46 μm, 6–7.5 μm wide (n = 4). Ascus apical plugs 0.5–1 μm high, 1.5–3 μm wide (n = 28), discoid, staining blue in Melzer’s reagent. Ascospores [7.0–] 9.1 (0.8) [–11.3] x [3.0–] 3.8 (0.4) [–5.9] µm (n = 130), ellipsoid, dark brown, with circumferential germ slit.

On *Fagus*; developing within the bark.

Specimens examined:

GERMANY. Westfalen, Münster, Gasselstiege, on *Fagus*, leg. F. v. Tavel (ZT Myc 0081856, duplicate ZT Myc 0081724 ex Herb. Volkart, sub *Anthostoma turgidum*); Sächsische Schweiz, am grossen Winterberge, 12 May 1889, on *Fagus*, leg. W. Krieger (ZT Myc 0081857, sub *Anthostoma turgidum*). PRINCIPALITY OF LIECHTENSTEIN. Ruggell, Halden, 440 m.a.s., 1 Jan 1990, on *Fagus sylvatica*, leg. J.P. Prongué, no. 900 (ZT Myc 0081880, sub *Lopadostoma turgidum*). ROMANIA. Moldava, Distr. Bacáu Slamic, 28 Aug 1933, on *Fagus sylvatica*, leg. Tr. Savulescu & S. Sandu (ZT Myc 0081860, sub *Anthostomella turgidum*). SPAIN. Vitoria, Gauna, 22 July 1981, on *Fagus*, leg. L. Petrini (ZT Myc 0081700, sub *Lopadostoma gastrinum*). SWITZERLAND. Canton Schwyz, Einsiedeln, 7 May 1978, on *Fagus sylvatica*, leg. G.J. Samuels (ZT Myc 0081863, sub *Lopadostoma turgidum*, mixed collection with *L. turgidum*); Canton Zurich, Zollikon, April 1949, on *Fagus sylvatica*, leg. E. Müller (ZT Myc 0081871, sub *Lopadostoma turgidum*); Canton Zurich, Zollikon, 20 May 1949, on *Fagus sylvatica*, leg. E. Müller (ZT Myc 0081870, sub *Lopadostoma turgidum*).

***Lopadostoma gastrinum*** (Fr.) Trav., Fl. ital. crypt. 1(2): 169 (1906). Figures 2G–L and 3B.

For a complete description and illustrations see Jaklitsch et al. (2014).

Stromata 2–5 × 2–4 mm wide, 1–3 mm high, round to elliptical in circumference, barrel shaped, black, with central disc, completely encasing the perithecia, stromatic line 75–200 μm thick. Ostiolar disc 0.6–2 mm diam., raising up to 0.3–0.4 mm above the bark, black, warty from perforating ostioles. Entostroma cream, light brown at base. Perithecia 375–875 μm wide, 650–925 μm high excl. ostioles (n = 11), up to eight per stroma, polystichous. Ostioles up to 2000 μm high, converging into disc. Asci total length 92–128 μm, spore bearing part 68–81 μm, stipe 23–47 μm, 9–9 μm wide (n = 10). Ascus apical plugs 0.5–1 μm high, 2–4 μm wide (n = 7), discoid, staining blue in Melzer’s reagent. Ascospores [10.0–] 11.6 (0.9) [–14.0] x [3.6–] 5.2 (0.6) [–6.3] µm (n = 120), ellipsoidal with broadly rounded ends and almost parallel sides, brown, with circumferential germ slit.

On *Abies alba* (?), *Acer, Ulmus*, other hardwood except *Fagus* and *Quercus.*

Specimens examined:

CZECH REPUBLIC. Moravě, Hranice (Mährig Weisskirchen), Olspitz, on *Ulmus campestris*, Aug 1912, leg. F. Petrak (ZT Myc 0081855, duplicate Z Myc 0011291). GERMANY. Münchau pr. Hattenheim, on *Ulmus campestris*, leg. Fuckel (ZT Myc 0081702, ZT Myc 0081703, Fungi rhenani 2005 *Quaternaria nitschkei*, sub 138 *Anthostoma gastrinum*, ex herb. Barbey-Boissier). SPAIN. Catalonia, Puerto de Bonaiga, 2000 m.a.s., on *Abies alba*?, wood, 24 May 1986, leg. F. Candoussau (ZT Myc 0081877). E: *ram. ulmei*, B: In *Acera campestri*, leg. Wallroth (STR, sub *Sphaeria succenturiata*).

Note: The stromata develop in the bark with ostiolar channels perforating the surface. If lower parts of the bark are degraded, stromata remain attached to the wood leaving empty spaces in between, as evident in specimen ZT Myc 0081877,

The specimen from Spain is considered to belong to *L. gastrinum*, because of its morphology and Jaklitsch et al. (2014) mention a specimen collected also by the French mycologist Françoise Candoussau at the same location eight days afterwards.

***Lopadostoma quercicola*** Jaklitsch, J. Fourn. & Voglmayr, Persoonia 32: 72 (2014).

For a complete description and illustrations see Jaklitsch et al. (2014).

Stromata 4–7 × 1.5–4 mm in diam, 1–2.5 mm high, round to elliptical in outline, discoid, black, with central disc, encasing the fructification completely, stromatic line 175–250 μm thick. Ostiolar disc 0.5–2 mm in diam., raising up to 0.6 mm above the bark, warty from perforating ostioles. Entostroma cream, wood-like. Perithecia 250–1000 μm in diam., 425–1075 μm high excl. ostioles (n = 41), pear shaped, laterally squashed, up to 15 per stroma, polystichous. Ostioles 500–1200 μm long, converging into disc, umbilicate, in age open, crater-like. Asci total length 100–165 μm, spore bearing part 58–111 μm, stipe 19–69 μm, width 6–11 μm (n = 40). Ascus apical plugs 0.5–1.5 μm high, 2–3 μm wide (n = 12), discoid, staining blue in Melzer’s reagent. Ascospores [7.2–] 11.6 (1.4) [–14.4] x [3.6–] 4.3 (0.4) [–5.4] µm (n = 140), broadly ellipsoidal with broadly rounded ends and almost parallel lateral sides, brown to dark brown, with circumferential germ slit.

On *Quercus*; developing between wood and bark, uplifting the bark.

Specimens examined:

AUSTRIA. Niederösterreich, Georgenberg bei Purkersdorf on *Quercus* sp., May 1949, leg. F. Petrak (ZT Myc 0081873, sub *Anthostoma gastrinum*). CZECH REPUBLIC. Moravě, Hranice (Mährig Weisskirchen), Svrčov, on *Quercus robur*, Oct 1926, leg. F. Petrak (ZT Myc 0081872, sub *Anthostoma gastrinum*). FRANCE. Landes (40), Lac de Léon, on *Quercus pedunculata*, 4 May 1986, leg. F. Candoussau (ZT Myc 0081878); Pyrénées Atlantiques, Sauveterre de Béarn, 28 Apr 1986, leg. F. Candoussau (ZT Myc 0081879); Landes, Tartas, Bord de la Midouze, 29 Mar 1986, leg. L. & O. Petrini, F. Candoussau, G. Gilles (ZT Myc 0081881); Pyrénées Atlantiques, Oloron, Île de Sauveterre, 28 Mar 1986, leg. L. & O. Petrini, F. Candoussau, G. Gilles (material no longer available). SWITZERLAND. Canton Zurich, Zurich, Wald am Zürichberg, on *Quercus*, 10 May 1937, leg. H. Schmid (ZT Myc 0081876, sub *Anthostoma gastrinum*); Canton Zurich, Zurich, Wald am Zürichberg, on *Quercus*, Feb 1937, leg. H. Schmid (ZT Myc 0081875, sub *Anthostoma gastrinum*); Canton Zurich, Zurich, Wald am Zürichberg, on *Quercus*, 20 May 1937, leg. H. Schmid (ZT Myc 0081874, sub *Anthostoma gastrinum*).

Note: Three specimens are without host indication. I assume it, however, to be *Quercu*s, as the collections are from a forest with mainly *Quercus* trees.

***Lopadostoma turgidum*** (Pers.) Trav., Fl. ital. crypt. 1(2): 170 (1906). Figure 2 M–Q, Fig. 3C.

For a complete description and illustrations see Jaklitsch et al. (2014).

Stromata 1–2 × 2–3 mm wide, 0.4–0.7 mm high, round to elliptical in outline, discoid, black, with central disc, encasing completely the fructification, stromatic line 25–50 μm thick. Ostiolar disc 0.1–0.5 mm in diam., oval to elliptical, warty from penetrating ostioles, at most 0.1 mm raising above the bark. Entostroma 25–50 μm thick around perithecia, dark grey, brown to black. Perithecia 225–1050 μm wide, 300–875 μm high excl. ostioles (n = 47), 3–12 per stroma, globose to cone-shaped. Ostioles 100–550 μm long, converging into disc. Asci total length 102–128 μm, spore bearing part 75–90 μm, stipe 20–28 μm (n = 5). Ascus apical plugs 0.5–1 μm high, 3–4 μm wide (n = 35), discoid, staining blue in Melzer’s reagent. Ascospores [8.6–] 10.4 (0.8) [–12.6] x [4.0–] 5.2 (0.3) [–6.0] µm (n = 160), ellipsoidal with broadly rounded ends and almost parallel lateral sides, dark brown, with unilateral, straight germ slit.

On *Fagus*; developing within the bark, ostioles hardly rising above.

Specimens examined:

AUSTRIA. Niederösterreich, Rotgraben bei Klosterneuburg, on *Fagus sylvatica*, May 1939, leg. F. Petrak (ZT Myc 0081867, sub *Anthostoma turgidum*). CZECH REPUBLIC. Moravě, Hranice (Mährig Weisskirchen), Ribar, on *Fagus sylvatica*, Aug 1940, leg. F. Petrak (ZT Myc 0081865, duplicate ZT Myc 0081864, sub *Anthostoma turgidum*). GERMANY. Munich, Pullach, on *Fagus sylvatica*, April 1893, leg. Schnabl (ZT Myc 0081854, sub *Anthostoma turgidum*); Hessen, in sylva Hostrichiensi, on *Fagus*, leg. Fuckel (ZT Myc 0081866, Rabenhorst,Fungi europaei no. 735, sub *Wuestneia sphinctrina*). SWITZERLAND. Canton Graubünden, Maienfeld, Steigwald, on *Fagus sylvatica*, 14 May 1986, leg. E. Müller (ZT Myc 0081701, sub *Lopdastoma gastrinum*); Canton Schwyz, Einsiedeln, on *Fagus sylvatica*, 07 May 1978, leg. G.J. Samuels (ZT Myc 0081863, mixed collection with *L. fagi*); Canton Zurich, Wald am Zürichberg, on *Fagus sylvatica*, Jan 1937, leg. H. Schmid (ZT Myc 0081869, sub *Anthostoma turgidum*); Canton Zurich, Sihlwald near Zurich, on *Fagus sylvatica*, Dec 1893, leg. F. v. Tavel (ZT Myc 0081862, duplicate ZT Myc 0081725 ex Herb. A. Volkart, sub *Anthostoma turgidum*); on *Fagus sylvatica*, Nov 1895, leg. Wegelin (ZT Myc 0081853, sub *Anthostoma turgidum*); Canton Zurich, on *Fagus sylvatica*, 15 Sep 1955, leg. E. Müller (ZT Myc 0081861, sub *Anthostoma turgidum*).

***Oligostoma insidiosum*** (P. Crouan & H. Crouan) Voglmayr, J. Fourn. & Jaklitsch, Persoonia 49: 91 (2022). Figures 2R–Y and 3D.

For a complete description and illustrations see Voglmayr et al. (2022).

Stromata 0.5–1.8 × 1–2 mm in diam., up to 1 mm high, elliptical in circumference, black, with central disc, covering perithecia like a shield, 25–100 μm thick, splitting top layer in several triangular lobes without uprising the substrate. Ostiolar disc 0.5–1.5 mm in diam., circular to elliptical in outline, dark brown to black, warty. Perithecia 300–600 μm wide, 350–750 μm high excl. ostioles. Ostioles 150–300 μm long (n = 7). Ascus apical plugs 4–7 μm high, upper width 5–7 μm, lower width 4–5 μm (n = 11), with bulky square upper rim, staining blue in Melzer’s reagent. Ascospores [18–] 25.5 (2.1) [–31] x [10.–] 11.4 (0.8) [–14] (n = 70), ellipsoidal, brown, with sigmoid germ slit, with 2–4 μm thick slimy sheath on ventral side.

Mainly on *Fagus sylvatica*, developing in the bark.

Specimens examined:

SWITZERLAND. Canton Vaud, supra Monthey, in *fagi ramis*, (ZT Myc 0081858, sub *Lopadostoma* (*Anthostoma*) *rhenanum*); Canton Zurich, Zürichberg, unweit Degenried, on *Acer platanoides*, Jan 1894, leg. F. v. Tavel (ZT Myc 0081717, sub *A. rhenanum*); Canton Zurich, Sihlwald bei Zürich, on *Fagus sylvatica*, 1 Nov 1893, leg. F. v. Tavel (ZT Myc 0081718, duplicate ZT Myc 0081719 ex herb Volkart, sub *A. rhenanum*); Canton Zurich, Zürich am Üetliberg unterhalb der Manegg, on *Fagus sylvatica*, Nov 1893, leg. F. v. Tavel (ZT Myc 0081720, sub *A. rhenanum*); Canton Zurich, Zürich, on *Fagus sylvatica*, Sep 1882, leg. G. Winter (ZT Myc 0081722 Rabenhorst Fungi Europaei 2870, duplicate ZT Myc 0081723, sub *A. rhenanum*); no indication, on *Fagus*, 05 Nov 1887, leg. Wegelin (ZT Myc 0081721, sub *A. rhenanum*).

**Fig. 4:**
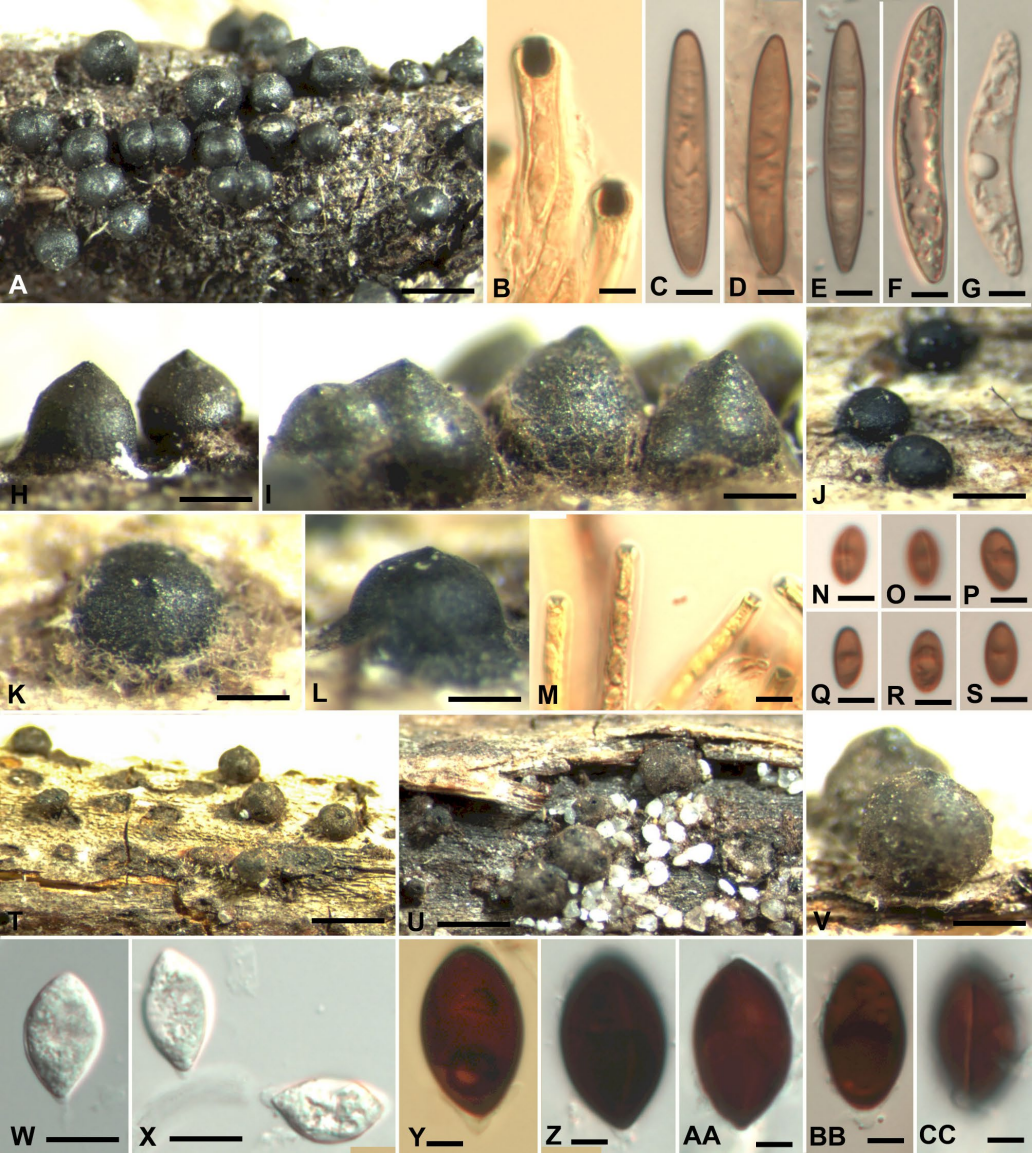
*Rosellinia mastoidiformis* (ZT Myc 0081883). **A, H, I**. Stromata. **B**. Ascus apical plugs in Melzer’s reagent. **C-H**. Ascospores, C showing germ slit. *Rosellinia neblinae* (ZT Myc 0081882). **J-L**. Stromata. **M**. Ascus apical plugs in Melzer’s Reagent. **N-S**. Ascospores, N, O showing germ slit. *Rosellinia schueppii* (holotype, ZT Myc 0081884). **T-V**. Stromata. **W, X**. Immature ascospores. **Y-CC**. Ascospores, Y-BB with cellular appendage, CC showing germ slit. Scale Bar: **A, T, U** = 1 mm. **H, J, V** = 0.5 mm. **I, K, L** = 0.25 mm. **B-G, M-S, W-CC** = 5 μm

***Rosellinia mastoidiformis*** Saccas, Agronomía trop. 11: 690 (1956). Figure 4 A–I.

Subiculum felted, brown, confined to stroma base. Stromata 575–725 μm high, 750–950 μm wide (n = 5), conical with integrated or poorly expressed ostioles, dark brown to black, in small groups, some stromata coalescent. Ectostroma 50 μm thick, perithecia attached. Ascus apical plugs 4–5 μm high, upper width 3.5–4 μm, lower width 3.5–4 μm (n = 5), inverted head-shaped with rounded upper rim, staining blue in Melzer’s reagent. Ascospores [29–] 34.8 (3.0) [–42] x [5–] 6.3 (0.8) [–8] µm (n = 30), long ellipsoidal, with broadly rounded ends and one flat side, light brown, pale, with indistinct straight germ slit, shorter than spore length. No slimy sheath or caps observed.

On corticated 0.3–0.5 cm diam. twigs.

Specimen examined:

SOUTH AFRICA. Mpumalanga, Lowveld, Nelspruit, Crocodile Valley Citrus Estates, on *Diospyros lycioides* Desf. (= *Royena lycioides* (Desf.) DC) ssp. *lycioides*, 16 Mar 1960, leg. H. Schüepp (ZT Myc 0081883).

Note: This material is morphologically similar as the type specimen collected on Manioc in the Central African Republic (Petrini 2013; Saccas 1956). As the ascospores are very light colored, the germ slit is difficult to observe, and it shows only as a faint lighter colored line.

***Rosellinia neblinae*** L.E. Petrini, Index Fungorum 25: 3 (2013). Figure 4 J–S.

Subiculum light brown, evanescent, reduced to stroma base. Stromata 250–440 μm high, 400–575 μm wide (n = 5), semiglobose to mammaeform, some surrounded by 50–80 μm wide stromatic ring, black, with finely papillate ostioles, singly. Ectostroma 50 μm thick. Perithecia detached. Ascus apical plugs 1–1.5 μm high, upper with 2–3 μm, lower width 1–1.5 μm (n = 5), fan shaped in cross section, staining blue in Melzer’s reagent. Ascospores [7.5–] 8.4 (0.5) [–9.5] x [4–] 4.5 (0.4) [–5] µm (n = 30), ellipsoidal, brown, with straight germ slit as long as spore.

On decorticated, heavily decomposed wood pieces.

Specimen examined:

PUERTO RICO. Bosque de Vega Alta west of S. Juan (Teak plantation); Playa de Dorado-Sardinera, Ruinas de Cañuela, Museo Ruinas de Caparra, on decorticated wood, 12 Jun 1988, leg. W. Gams (ZT Myc 0081882).

Note: This specimen shows the typical stromal shape of a *Rosellinia* and has an evanescent subiculum still visible around some stromata. The stromatic ring probably serves to anchor the stroma, as the substrate is very crumbly. It keys out as *R. neblinae* and its morphology agrees with the description of the type originating from Venezuela (Petrini 2013).

***Rosellinia schueppii*** L.E. Petrini, sp. nov. Figure 4T–CC.

Index Fungorum ID: IF 901140.

**Typification. SOUTH AFRICA** Cape Peninsula, Hout Bay, Noordhoek Peak, on *Myrcia cordifolia*, 16 Nov 1959, leg. H. Schüepp (holotype of *Rosellinia schueppii*, ZT Myc 0081884).

**Etymology** According to the collector Hannes Schüepp, late plant pathologist at the Swiss Federal Research Station, Wädenswil, who was a postdoctoral researcher at University of Pretoria.

Subiculum felted, dark brown, reduced in extension, not present on bark, only in areas where bark was peeled away. Stromata 800–1200 μm high, 950–1500 μm wide (n = 5), subglobose, apically slightly compressed with blunt ostioles, dark brown, single or in small groups. Ectostroma 75 μm thick. Perithecia detached. Ascospores [20–] 23.5 (1.5) [–28] x [11–] 13 (1) [–15] µm (n = 30), lemon-shaped, dark brown, with one semiglobose cellular appendage, with a straight germ slit as long as the spore.

On bark or in ruptures of bark, on 0.5–1 cm diam. twigs.

Specimen examined:

SOUTH AFRICA. Cape Peninsula, Hout Bay, Noordhoek Peak, on *Myrcia cordifolia*, 16 Nov 1959, leg. H. Schüepp (holotype, ZT Myc 0081884).

Note: The ascus apical plug was not observed. Lemon-shaped ascospores characterize this species and differentiate it from *R. bonaërensis* Speg., *R. corticium* (Schw. : Fr.) Sacc., and *R. caudata* Petch, all three with overlapping stroma and ascospore size but with a much more abundant and persistent subiculum and distant geographical distributions (Petrini 2013). *Rosellinia medullaris* (Wallr.) Ces. & De Not. is also morphologically close to this South African specimen for stroma and ascospore shape but has smaller ascospores and stromata as well as a different geographic origin (Petrini 2013). Additional species having ascospores with appendages or slimy sheath and overlapping length are *R. granulosa* J. Fourn. & Lechat and *R. truncatispora* J. Fourn. & Lechat (Fournier et al. 2017), *R.yuannanensis* Wei Li bis & L. Guo (Li and Guo 2015), as well as *R. angusta* Wei Li bis & L. Guo (Li and Guo 2019), but they differ from *R. schueppii* by ascospore width and shape. The erection of the new species is based on morphology. Distinct ascospore shape combined with size and stroma characters are sufficient to formally name this yet undescribed *Rosellinia* to make mycologists aware of its existence.

As the nomenclatural code does not permit mutated vowels, the epithet “schüeppii” had to be registered as “schueppii”.

## Discussion

Thanks to the monographic treatment of *Lopadostoma* (Jaklitsch et al. 2014) the ZT specimens collected between 1889 and 1990 could be named. In the past, to identify either *L. turgidum* or *L. gastrinum*, the host had priority over ascospore morphology, difficult to examine in detail with the optical means available at that time. *Lopadostoma fagi* was as frequently misidentified as *L. turgidum*. Both species seem to be equally frequent in *Fagus sylvatica* forests, as documented also by a mixed collection of *L. fagi* and *L. turgidum* (ZT Myc 0081863) and same collecting sites in Austria (Jaklitsch et al. 2014). The host has gained again priority to separate morphologically and ecologically *L. gastrinum* from *L. quercicola* as differences in stromal characters are subtle, ascospore size is overlapping and differences in molecular phylogeny can be detected only in well-equipped laboratories.

Presently, the identification of *R. mastoidiformis* and *R. neblinae* is based on morphology alone. Interestingly, the second specimens were found in the same greater area as the type. It is important to be able to record an additional specimen of each *R. mastoidiformis* and *R. neblinae*, which are so far known only from the type material and to describe a new species of *Rosellinia*, 64 (for *R mastoidiformis* and *R. schueppii*) and 35 years (for *R. neblinae*) after the material was collected.

## Conclusion

This publication relies on specimens collected in the past, some of them over 100 years ago. Contributions to the content of an herbarium by mycologists and individuals interested in nature were and are still fundamental (Watling 1998; Webster 1997) as they represent an invaluable source of information. This publication illustrates on the one hand the importance of providing good identification keys in monographic works such as those by Jaklitsch et al. (2014) and Voglmayr et al. (2022), and on the other, that the study and revision of herbarium material may lead to surprising discoveries that not only justify but also make essential keeping fungal collections.

## Data Availability

The datasets generated during and/or analyzed during the current study are available from the author upon reasonable request. All material but one specimen (STR) is deposited in the herbarium ZT.
